# The effect of lizards on the dispersal and germination of *Capparis spinosa* (Capparaceae)

**DOI:** 10.1371/journal.pone.0247585

**Published:** 2021-02-26

**Authors:** Yi Yang, Yingying Lin, Lei Shi

**Affiliations:** College of Animal Science, Xinjiang Agricultural University, Urumqi, Xinjiang, China; Chinese Academy of Forestry, CHINA

## Abstract

Seed dispersal is a key component of the interactions between plants and animals. There is little research on the effects of lizard seed dispersal, which is more common on islands than elsewhere. In this study, the effects of the passage of *Capparis spinosa* seeds through *Teratoscincus roborowskii* lizard digestive tracts on the seed coats, water uptake rates and germination rates were investigated. In addition, the spatial patterns of fecal deposition by lizards in various microhabitats were assessed. Our results showed that the mean retention time (MRT) of mealworms was significantly longer than that of *C*. *spinosa* seeds in both adult and juvenile lizards. The defecation rate of *C*. *spinosa* tended to be lower than that of mealworms, which might be beneficial for seed dispersal. It was determined that the longer MRT of *C*. *spinosa* seeds enhanced the permeability of the seed coats, which promoted fast water uptake, broke seed dormancy and increased the seed germination rate. Furthermore, the seeds that passed through the digestive tracts of lizards were deposited in favorable germination microhabitats. By enhancing seed germination and depositing intact and viable seeds in safe potential recruitment sites, the lizard *T*. *roborowskii* acts, at least qualitatively, as an effective disperser of *C*. *spinosa*.

## Introduction

Seed dispersal is a key component of the interactions between plants and animals [1,2]. The study of frugivorous behavior in animals and of plant interactions has been critical in the development of both ecological and evolutionary theories. The dispersal of many plants depends on transport by seed-dispersing animals [3]. A growing body of research has reported seed dispersal mediated by fish [4], birds [5], primates [6], turtles [7] and lizards [8], which are potential seed dispersers. Lizards can be effective seed dispersers in terms of fruit removal, seed deposition in suitable habitats and seed germination [9]. However, there is little research on the effects of seed dispersal by lizards, which is more common on islands than elsewhere [10].

The effectiveness of seed dispersal depends on the quantity and quality of seed dispersal [11]. The quantity of seed dispersal depends not only on the number of visits made to a plant by a disperser but also on the number of seeds dispersed per visit [12]. However, the quantitative (i.e., the number of visits or number of dispersed seeds) and qualitative (i.e., increasing dispersal distance, enhancement of seed germination success, and delivery of seeds at sites whose characteristics may favor seed and seedling survival) attributes of dispersal vary in effectiveness among species or functional groups [11].

The retention time of food in the digestive tract is one of the most important factors affecting seed dispersal quality because it determines the length of time that seeds are exposed to potentially beneficial or destructive digestion processes and affects the spatial scale and pattern of seed deposition [13]. There is a trade-off between the factors that affect the effectiveness of seed dispersal. A long retention time increases the probability of long-distance dispersal, which is a favorable feature for plants but may reduce seed vitality. It was found that the seed germination rate decreased with increasing food retention time in the digestive tract of the tortoise *Aldabrachelys gigantea*, which reduced the dispersal advantage [14]. Digesta retention time can be measured based on the following three indicators: Time of first appearance (TFA), mean retention time (MRT) and time of last appearance (TLA) [15]. Food intake may influence digesta retention time; for example, with an increase in the number of fruits consumed, the retention time is reduced. In fruit-eating birds, to increase the rate of digestion, the MRT also tends to be shorter [16].

The effectiveness of seed dispersal is greatly influenced by the interactions between seeds and the digestive tracts of animals [17]. The retention time of food in the digestive tracts of animals has important implications for digestive physiology [13]. For several fleshy-fruited plants, passage of seeds through the digestive tracts of frugivores enhances the germination of the seeds. Reptiles, including turtles and lizards, have been reported to affect seed germination in various kinds of plants [10,13]. It has been shown that reptiles could affect the rate of germination in most cases (63%), of which the promotion of seed germination was observed in 47% of cases and the inhibition of seed germination was observed in 16% of cases [18]. It was shown that seeds of *Solanum thomasiifolium* that were digested by lizards had a germination rate of 80%, which was higher than the germination rates after digestion by birds (64%) and foxes (53%) [19]. To cope with seasonal variation in food availability, animals often alter their feeding behavior, such as their food preferences, to satisfy their nutritional requirements.

Lizard frugivory has been reported in a variety of environments, such as Mediterranean-type climate ecosystems, temperate rainforests and high-elevation Andes shrubs. Seed dispersal by lizards has been described as a typical island phenomenon [20]. Our previous study showed that 85% of the total biomass consumed by *Teratoscincus roborowskii* was *Capparis spinosa*, suggesting that *C*. *spinosa* may play a crucial role in the adaptation of *T*. *roborowskii* to the extremely arid environment in the Turpan Basin and that the feeding behavior of *T*. *roborowskii* may have an impact on *C*. *spinosa* seed dispersal. *C*. *spinosa* belongs to the Capparaceae family that is mainly distributed in the Xinjiang Uygur Autonomous Region [21]. It is a dicotyledonous perennial shrub and has extensive root systems, which protects soils from erosion and beneficial to desertification control. Our previous study found that the larger *C*. *spinosa* fruit became increasingly elliptical, with width increasing more slowly than length, which might be the result of the selection pressure of the gape width of *T*. *roborowskii* [22]. However, it is difficult to propagate seedlings because of germination problems due to dormancy and hard seeds of *C*. *spinosa* [23]. *T*. *roborowskii* is an endemic species that is only distributed in the Turpan Depression of the Xinjiang Uyghur Autonomous Region, China. The investigation of *T*. *roborowskii* has mainly focused on mimicry [24], foraging modes [25], activity rhythm [26], sexual dimorphism, diet, skeletochronology [27], home range [28], habitat [29] and food chemical discrimination [30]. However, the effect of *T*. *roborowskii* on the germination and dispersal of plants has rarely been reported. Due to the special frugivorous behavior of *T*. *roborowskii*, we address the following questions. (1) What are the effects of passage through the digestive tract of *T*. *roborowskii* on the *C*. *spinosa* seed coat, water uptake and germination? (2) Is seed deposition via fecal material congruent with the proportional availability of microhabitats in the environment inhabited by this lizard species? In the present study, the digesta retention time (DRT) of *C*. *spinosa* seeds in *T*. *roborowskii* and the germination of seeds with different DRTs were investigated to evaluate the implication of *T*. *roborowskii* on *C*. *spinosa* seed dispersal.

## Material and methods

### Study site and animals

**Fig 1:**
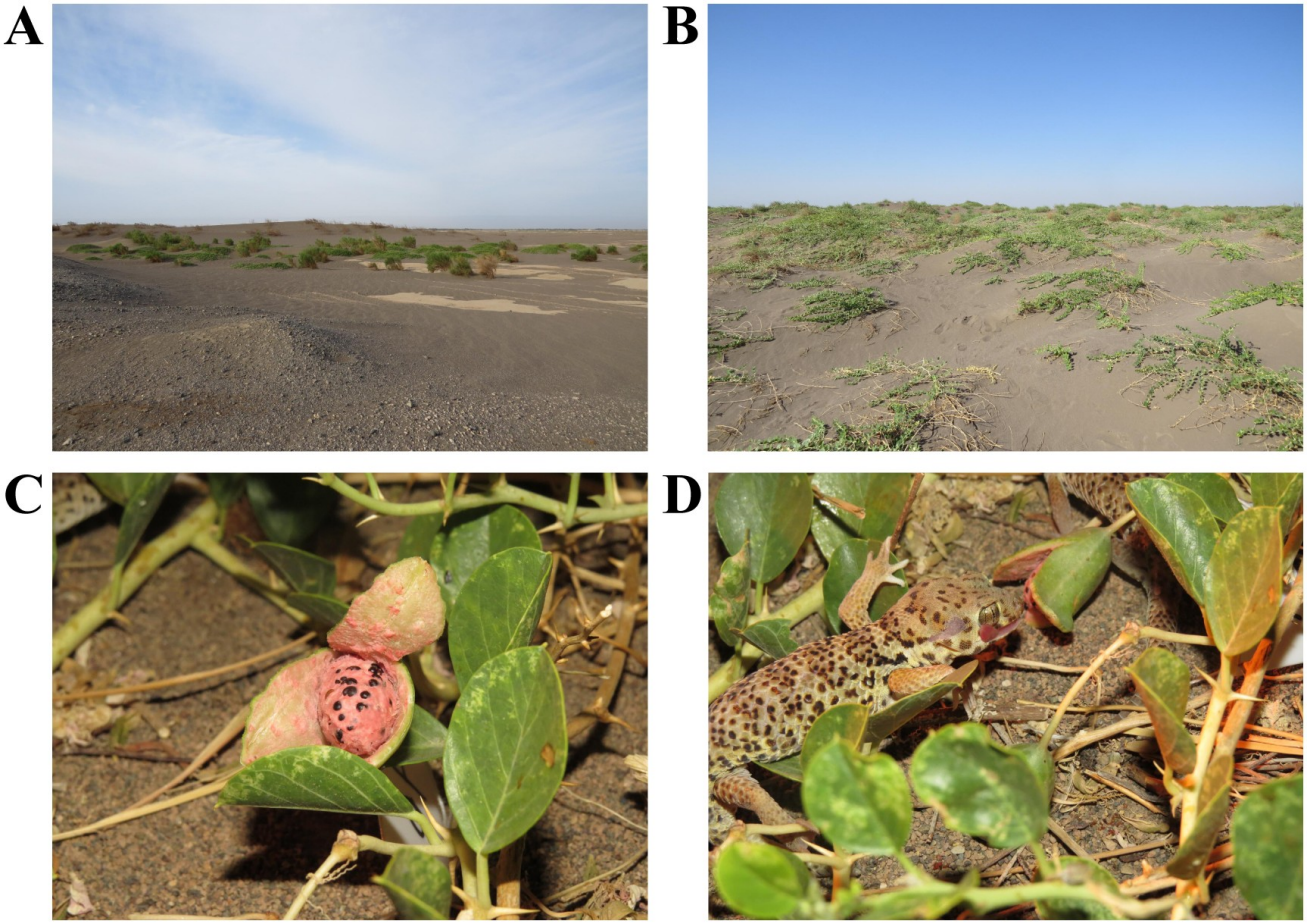
Study area in the Turpan Eremophytes Botanic Garden at the Chinese academy of sciences in the Turpan Basin of northwest China’s Xinjiang Uygur Autonomous Region. (A) Distant view of sample site. (B) Close-up view of sample site. (C) Ripe fruit of *C*. *spinosa*. (D) *T*. *roborowskii* feeding on the fruit of *C*. *spinosa*.

Eighty-two *T*. *roborowskii* (thirty-two males and fifty females) were captured at the Turpan Eremophytes Botanic Garden at the Chinese Academy of Sciences (E89°11′, N42°54′) in August 2014 (Fig 1). This study was conducted in compliance with current laws regarding animal welfare and research in China and the regulations set by Xinjiang Agricultural University. All experimental procedures involving animals were approved by the Animal Welfare and Ethics Committee of Xinjiang Agricultural University, Urumqi, Xinjiang, China. After the experiment was completed, all lizards were released to the Turpan Eremophytes Botanic Garden.

### Feeding experiments

Feeding experiments with captive animals are the traditional method for obtaining the retention time of food in the digestive tract of animals. The lizards were divided into two groups according to their snout-anus lengths (SVL): the adult group (SVL > 74.77 mm) and the juvenile group (SVL < 74.77 mm). Fifty-two adult lizards and 30 juvenile lizards were numbered and housed in breeding boxes with free access to vitamin water. After 15 days, lizards were given free access to peeled *C*. *spinosa* for 3 h according to their usual schedule (after 21:30). Then, the uneaten *C*. *spinosa* was weighed, and the food intake of each lizard was calculated.

### DRT

In captivity, some lizards do not eat due to having left their living environments. Among the 52 adult lizards and 30 juvenile lizards, the 14 lizards in each group with the highest feed intakes were selected with which to further explore the digesta passage time. Fecal samples were collected at 01:00, 07:00, 13:00 and 19:00 every day for up to 15 days. A digital camera was used to observe the defecation events, and the defecation time of each lizard was obtained by video.

The MRT, which refers to the mean digesta passage time of *C*. *spinosa* seeds from ingestion to excretion, was calculated according to the following equation:

$$\text{t}=\overset{n}{\underset{i=1}{\sum }}m_{i}t_{i}/\overset{n}{\underset{i=1}{\sum }}m_{i}$$

where *m*_*i*_ is the number of *C*. *spinosa* seeds excreted at time *t*_*i*_ after feeding.

When the *C*. *spinosa* feeding experiment was finished, the lizards were given free access to animal-based food, i.e., mealworms, with the same protocol and analysis method.

### Excretion rate

The newly produced feces of each lizard was collected immediately, and the defecated seeds were cleaned using distilled water. Then, the separated seeds were counted and dried with absorbent paper and stored individually in a dry place at room temperature. The excretion rate and cumulative excretion rate were calculated using the following formulas:

Excretionrate(%)=numberofdefecatedseeds/totalnumberofdefecatedseeds×100%.

Cumulativeexcretionrate(%)=numberofcumulativedefecatedseeds/totalnumberofdefecatedseeds×100%.

In the mealworm feeding experiment, the residues of mealworms in feces, including the exoskeletons and mouthparts of the mealworms, were separated, air dried and weighed. The excretion rate and cumulative excretion rate were calculated using the following formulas:

Excretionrate(%)=weightofdrymatterperdefecatedmaterial/totalweightofdrymatterindefecatedmaterial×100%.

Cumulativeexcretionrate(%)=weightofdrymatterincumulativedefecatedmaterial/totalweightofdrymatterindefecatedmaterial×100%.

### Calculation of defecation frequency

The defecation events of each lizard in the adult and juvenile groups were counted every 24 h by observing the videos. The defecation rates were calculated according to the following equation:

Defecationrate(times/day)=totaldefecationtime/totalobservationdays

### Scanning electron microscopy (SEM)

Seeds separated from feces with DRTs of 0–72 h, 72–120 h and 120–360 h were used as treatment groups, and seeds separated from fresh fruits were used as control groups. All the dried seeds were completely gold-sputtered under vacuum conditions. Scanning electron microscopy images were collected with magnifications of 80×, 500× and 3000× (LEO-1430VP, Carl Zeiss, Germany).

### Seed water uptake detection

The seeds that were separated from feces were used as treatment groups, and seeds separated from fresh fruits were used as control groups. The water uptake of *C*. *spinosa* seeds (n = 160) was determined gravimetrically at 2 h, 4 h, 6 h, 8 h, 10 h and 12 h, and the surface water was removed with tissue paper. An electric balance was used to weigh the seeds, and then the seeds were returned to the dishes and reweighed at the scheduled time. The increase in the weight of the seeds indicated the amount of water uptake. The water uptake percentage was calculated using the following formula:

$$\text{Water}\;\text{uptake}\;\text{percentage}\;(\%)=({\text{m}}_{1}-{\text{m}}_{0})/{\text{m}}_{0}\;\times \;100\%$$

where m_0_ is the weight of seeds before water uptake and m_1_ is the weight of seeds after water uptake.

### Seed germination and viability experiment

A germination experiment was used to detect whether the DRT affected seed germination. Seeds separated from feces and fresh fruit were both precooled at 4°C for 60 days and then transferred to a germination experiment in a 25°C incubator for 30 days. The germination rate was estimated as a ratio between the number of germinated seeds and the total number of seeds per dish. Each seed was determined to have germinated when a radical sprout with a length of 1 mm appeared from the seed. The germination rate was calculated according to the following equation:

Germinationpercentage(%)=seedsgerminated/totalseeds×100%

The viability of seeds that did not germinate was assessed by staining with 2,3, 5-triphenyl tetrazolium chloride (TTC). The seeds were placed in a dish containing a TTC aqueous solution (0.6 w/v in 0.05 M phosphate buffer) and incubated for 24 h at 25°C in the dark. The stained seeds were then examined under a stereomicroscope. The area of each seed that was stained red was considered viable, while the unstained seeds were considered nonviable. The seed viability was calculated according to the following equation:

Seedviability(%)=(seedsviable+seedsgerminated)/totalseeds×100%

### Fecal deposition and new seeding sites

To assess whether lizard feces are deposited in various microhabitats in proportion to their availability at the study site, the percentage cover of various microhabitats in the study area was recorded. Eleven 50 m × 1 m parallel transects were established, and 8 microhabitat categories that were present in the environment were arbitrarily defined: (1) *C*. *spinosa*, (2) *Alhagi sparsifolia*, (3) *Zygophyllum fabago*, (4) *Calligonum spp*., (5) *Karelinia caspia*, (6) deadwood, (7) dead grass, and (8) clearing. Within each transect, the microhabitats of all lizard fecal depositions (172 fecal samples) and new seeding sites (12 seedling samples) were recorded. The Vanderploeg selection coefficient *W*_i_ and the Scavia selection index *E*_i_ were used to evaluate the location preferences of *T*. *roborowskii* fecal depositions and *C*. *spinosa* seedlings for habitat selection. The calculations were completed according to the following equations:

$${\text{W}}_{i}=(r_{i}/p_{i})/\Sigma (r_{i}/p_{i})$$

$${\text{E}}_{i}=({\text{W}}_{i}-1/n)/({\text{W}}_{i}+1/n)$$

where W_*i*_ is the selection coefficient, E_*i*_ is the selection index, *i* refers to a specific environmental characteristic, *r*_*i*_ is the number of quadrats containing the *i*^th^ characteristic that is selected by the species, *p*_*i*_ is the total number of quadrats containing the *i*^th^ characteristic, and *n* is the number of grades of a specific environmental characteristic (*n* = 1, 2, …, n). When E_*i*_ = 1, the species has a particular preference; when E_*i*_ = -1, there is no selection preference; when E_*i*_ < -0.1, there is a negative selection preference; when E_*i*_ > 0.1, there is a positive selection preference; and when E_*i*_ = 0, there is random selection. There is also considered to be random selection when -0.1 ≤ E_*i*_ ≤ 0.1.

### Statistical analysis

Data are shown as the mean ± the standard error of the mean. The statistical analysis was performed by one-way analysis of variance (ANOVA). The two-tailed paired t test was used to compare the adult and juvenile groups and the intake rates of mealworms and *C*. *spinosa* in the adult and juvenile groups. A chi-square test was performed to assess the different spatial deposition patterns of feces (percentage of feces deposited in each microhabitat) and seedlings and the availability of the 8 microhabitats at the study site. *P* < 0.05 was considered to be statistically significant.

## Results

### Food intake and digesta retention time

**Fig 2:**
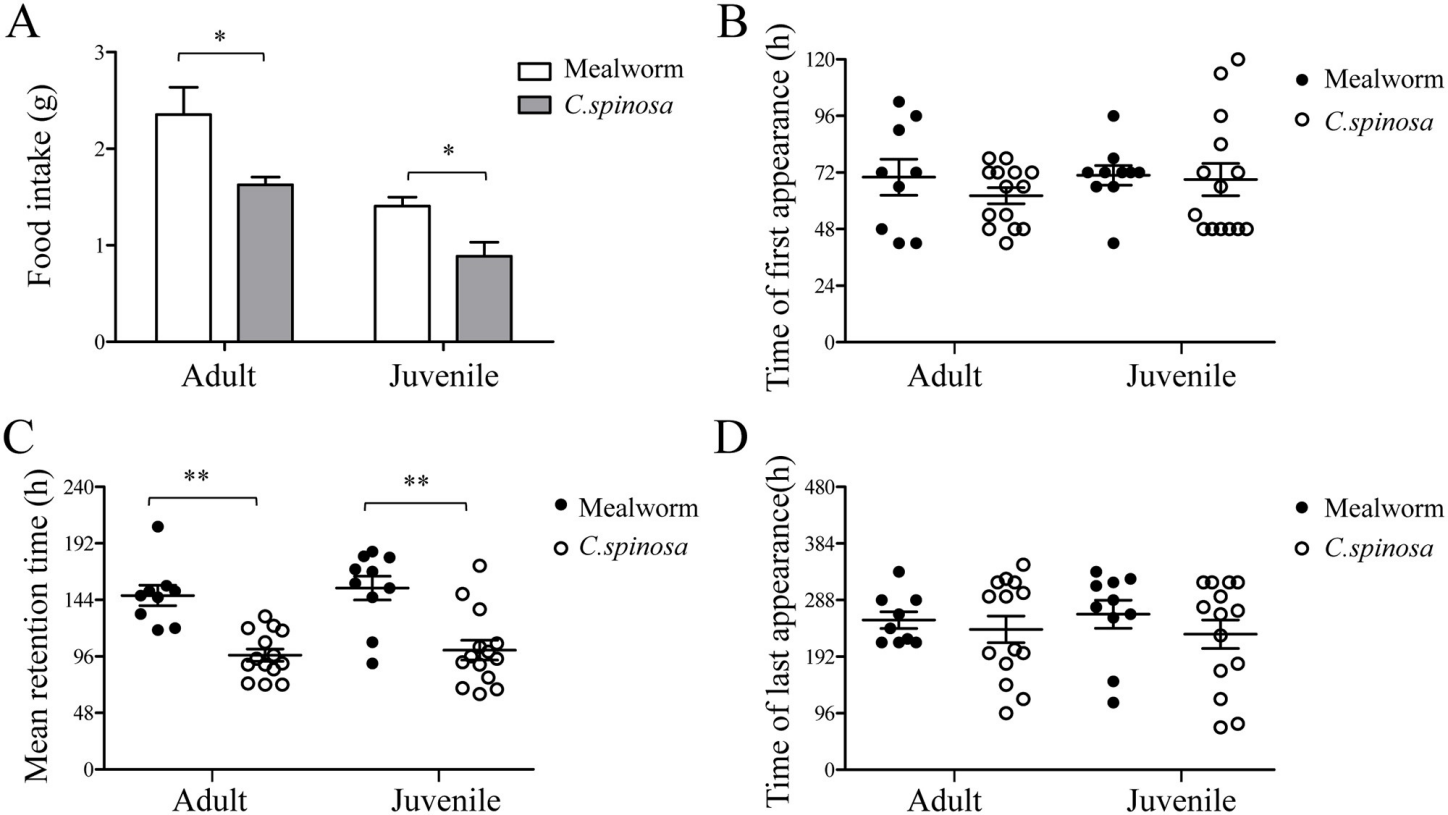
Effects of age and food type on food intake and digesta retention time in adult and juvenile lizards. (A) The intake of mealworms and *C*. *spinosa* in adult and juvenile lizards. (B-D) The TFAs, MRTs and TLAs of mealworms and *C*. *spinosa* in adult and juvenile lizards. The data were analyzed by a two-tailed paired t test. * *P* < 0.05; ** *P* < 0.01.

Lizards of both the adult and juvenile groups consumed relatively more mealworms than *C*. *spinosa* (*P* < 0.05). As adult lizards require more calories, the two experimental groups displayed the same feeding patterns, but the intake values of mealworms and *C*. *spinosa* in the adult group were significantly higher than those in the juvenile group (*P* < 0.05) (Fig 2A).

*C*. *spinosa* remained in the lizard gut for a range of 42 h to 348 h, and the retention times of mealworms were 42 h to 336 h. As shown in Fig 2B and 2D, age and food type did not remarkably affect TFA or TLA in lizards (*P >* 0.05). Furthermore, the MRT of mealworms and *C*. *spinosa* was not significantly different between adult and juvenile lizards (*P >* 0.05); however, the MRT of mealworms was significantly longer than that of *C*. *spinosa* in both the adult and juvenile groups (*P* < 0.01) (Fig 2C).

### Effects of age and food type on excretion rate

**Fig 3:**
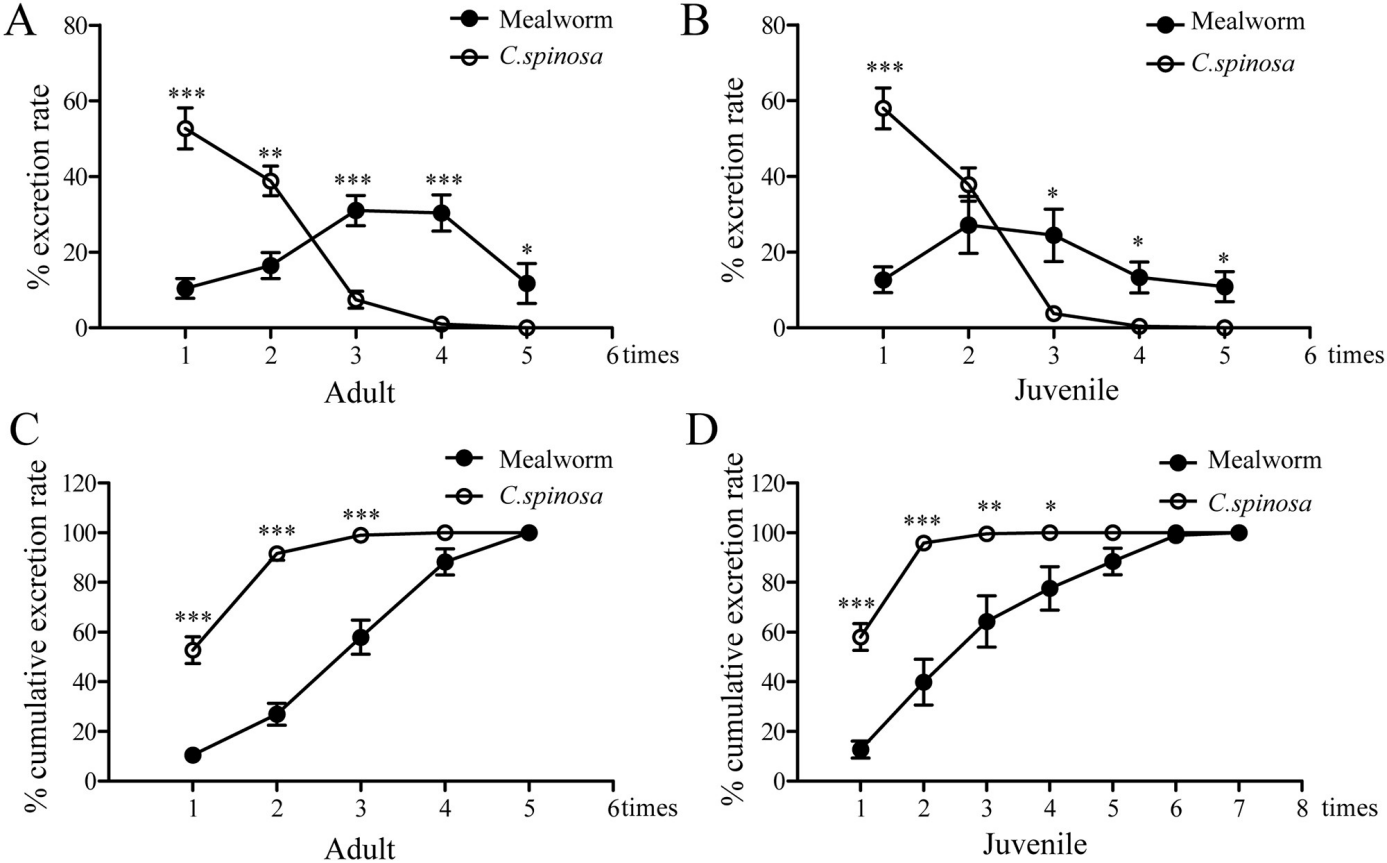
Effects of age and food type on excretion rate and cumulative excretion rate in adult and juvenile lizards. (A, B) The excretion rates of mealworms and *C*. *spinosa* in adult and juvenile lizards. (C, D) The cumulative excretion rates of mealworms and *C*. *spinosa* in adult and juvenile lizards. The data were analyzed by a two-tailed paired t test. * *P* < 0.05; ** *P* < 0.01; *** *P* < 0.001.

As shown in Fig 3, no significant differences were found between adult and juvenile lizards in the excretion rates and cumulative excretion rates. The excretion rate peak of mealworms always occurred at the first defecation event in both adult and juvenile lizards, and then decreases in the excretion rate were observed in the remaining defecation events (Fig 3A and 3B). However, the excretion rates of the first two defecation events reached 90%, and the cumulative excretion rate reached 100% in the 4th defecation event. In contrast to mealworm excretion, the excretion rate of *C*. *spinosa* was clearly increased in the 3rd and 4th defecation events. Particularly in juvenile lizards, the cumulative excretion rate reached 100% after seven defecation events (Fig 3D).

### Effects of age and food type on defecation frequency

**Fig 4:**
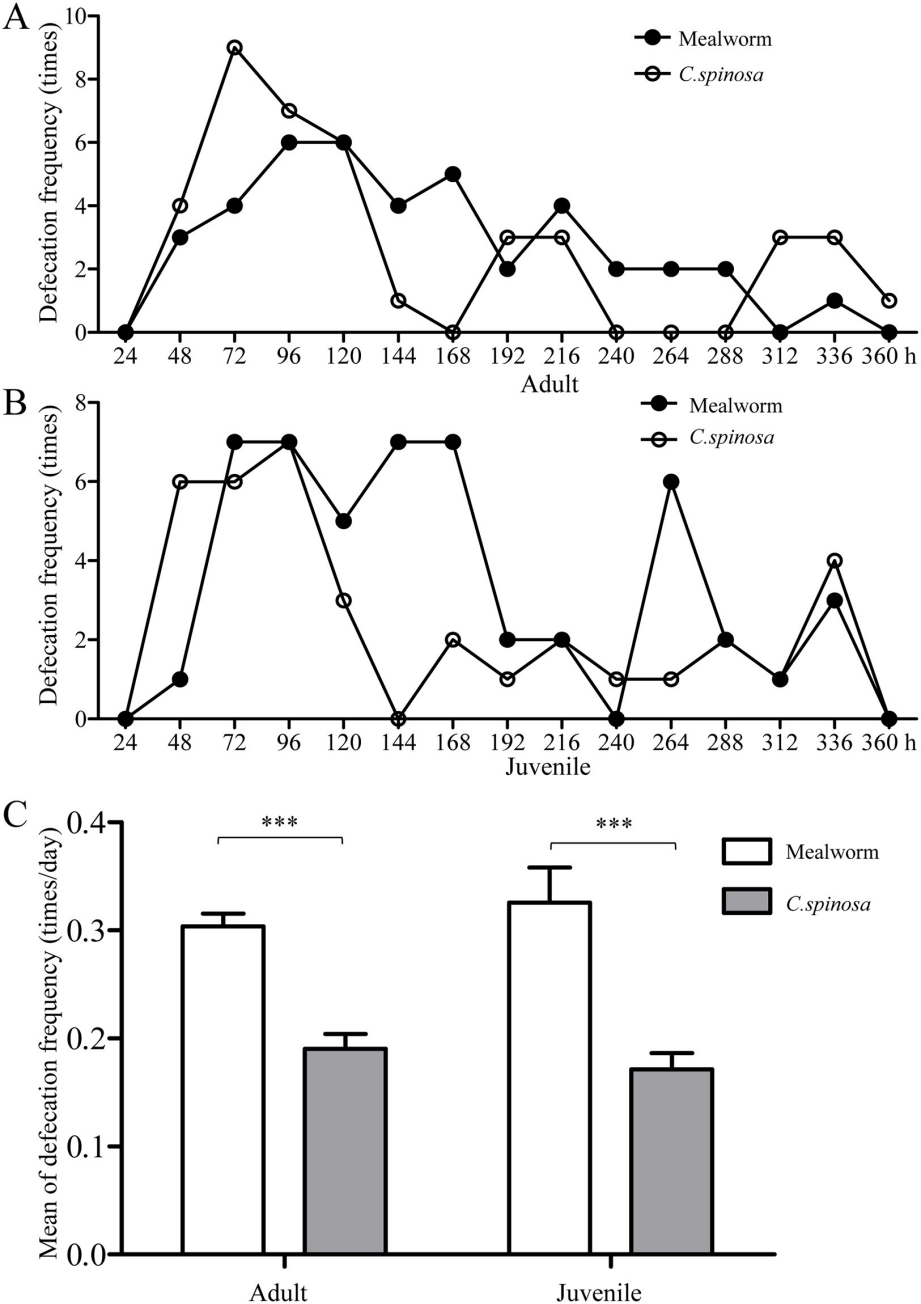
Effects of age and food type on defecation frequencies in adult and juvenile lizards. (A, B) The defecation frequencies of mealworms and *C*. *spinosa* in adult and juvenile lizards. (C) The defecation rates of mealworms and *C*. *spinosa* in adult and juvenile lizards. The data were analyzed by a two-tailed paired t test. *** *P* < 0.001.

In the adult lizard group, 41 (mealworms) and 40 (*C*. *spinosa*) defecation events were recorded over 360 h of observation (Fig 4A). Fifty (mealworms) and 36 (*C*. *spinosa*) defecation events were observed in the juvenile group (Fig 4B). However, the defecation patterns of mealworms were notably different from those of *C*. *spinosa* in both adult and juvenile lizards. During the observation period, the defecation frequency of *C*. *spinosa* in both adult and juvenile lizards was obviously increased at 0–120 h, while the defecation frequency of mealworms was concentrated in the first 5 days.

As shown in Fig 4C, the defecation rate of *C*. *spinosa* tended to be lower than that of mealworms (*P* < 0.001), which was approximately equal to 0.3 defecation events per lizard per day.

### Seed coat morphology

**Fig 5:**
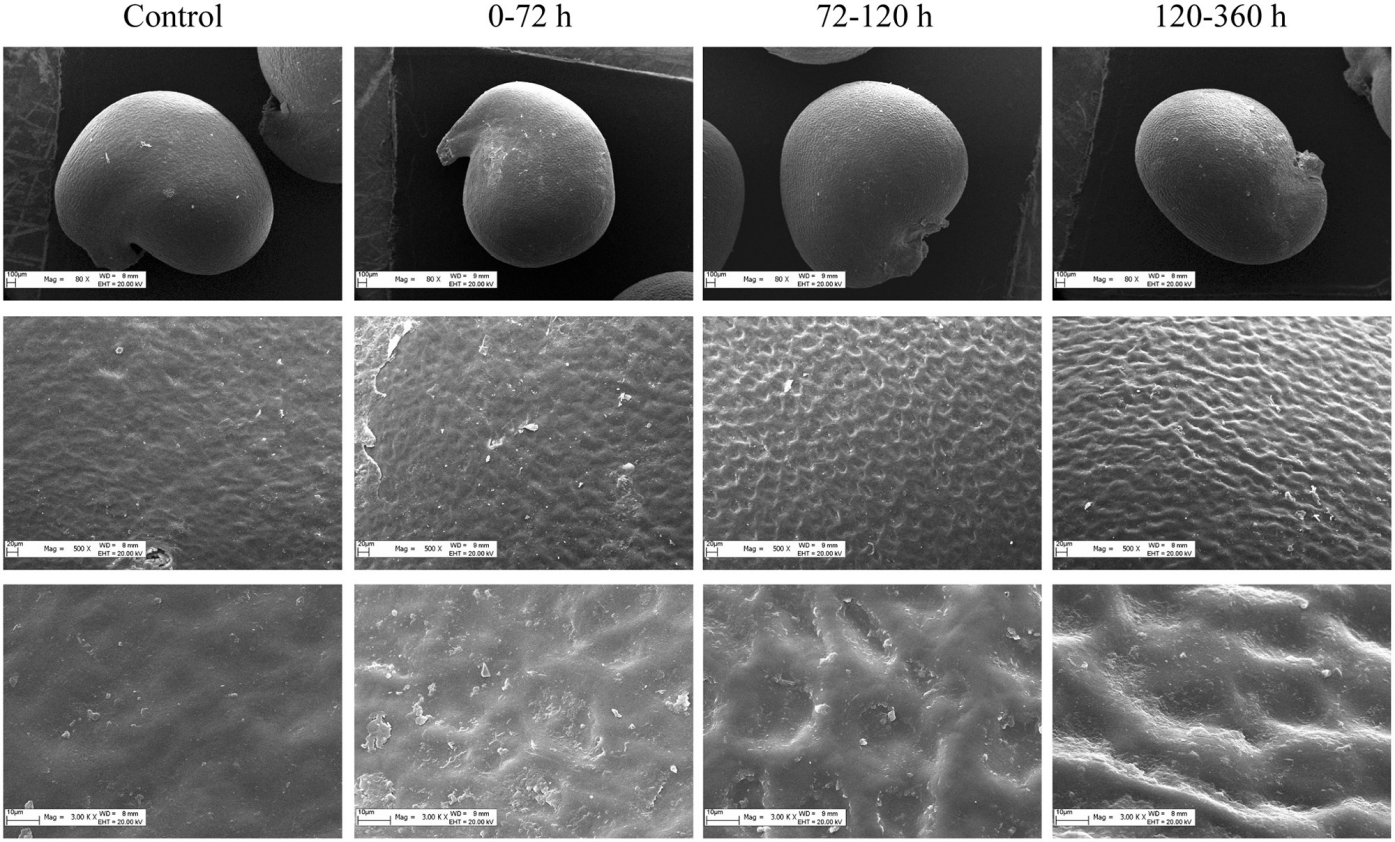
Effect of the DRT on *C*. *spinosa* seed coat morphology. The morphological changes of *C*. *spinosa* seed coats with different DRTs were observed by SEM with images magnified to 80×, 500× and 3000×.

Compared with the control group, the morphology of the seed coats changed notably in a time-dependent manner, characterized by seed coat corrugation and sunkenness with prolonged retention time (Fig 5).

### Water uptake

**Fig 6:**
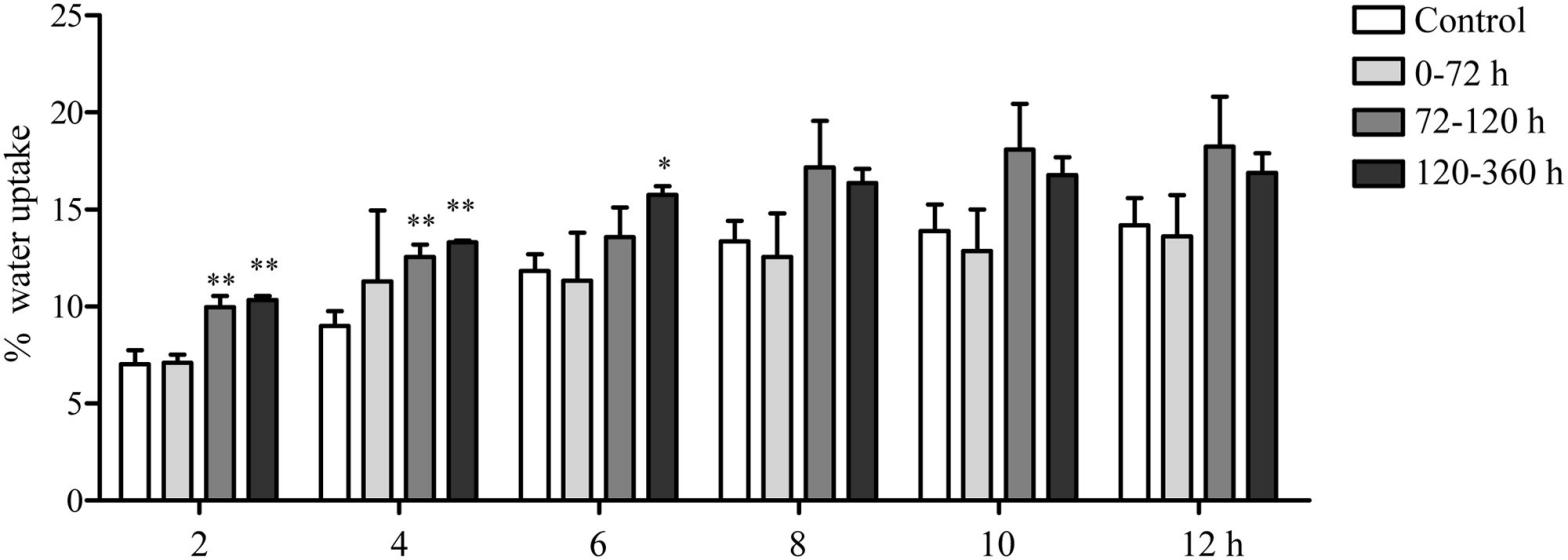
Effect of the DRT on *C*. *spinosa* seed water uptake. Water uptake of *C*. *spinosa* seeds with different DRTs. The data were from 3 independent experiments and were analyzed by ANOVA. * *p* < 0.05; ** *p* < 0.01 compared to the control group.

Compared with the control group, the DRT of 0–70 h had no significant effect on the water uptake of seeds (*P >* 0.05) (Fig 6). In contrast, the seed water uptakes after DRTs of 72–120 h and 120–360 h were significantly increased after 2 h and 4 h of water absorption (*P* < 0.01) compared with the water uptakes of the control group. Moreover, it was observed that the water uptake capacity of *C*. *spinosa* seeds improved slowly with increasing time.

### Seed germination and viability

**Fig 7:**
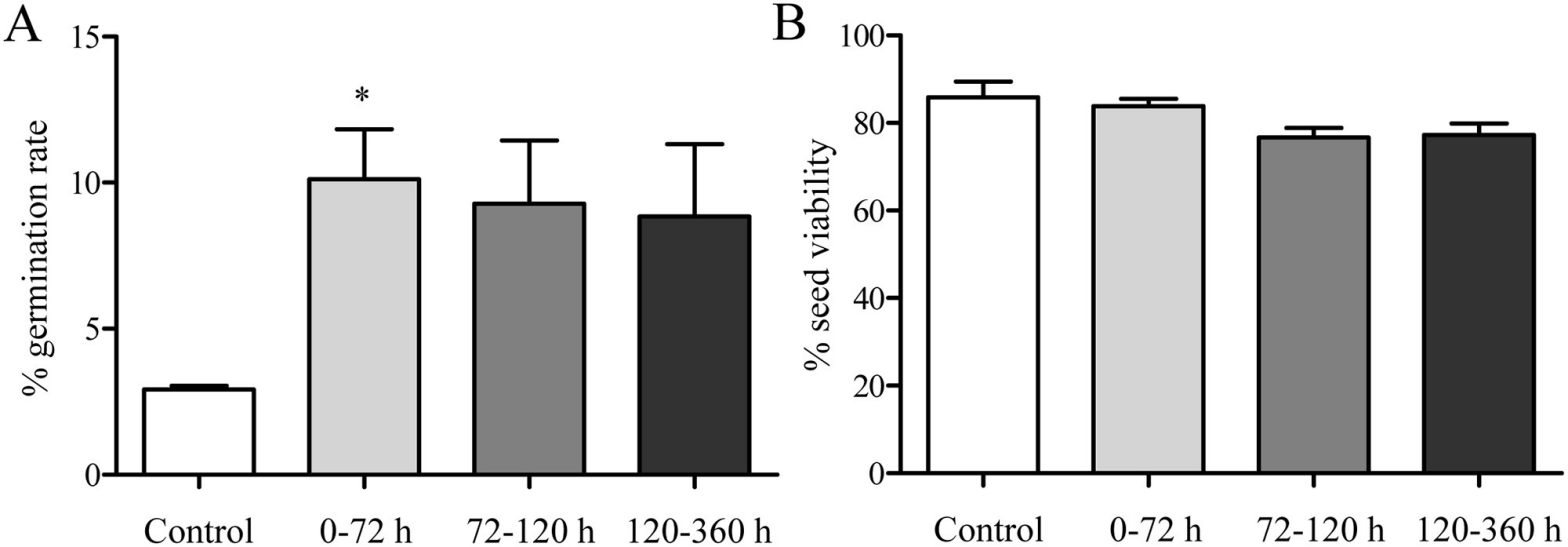
Effect of the DRT on *C*. *spinosa* seed germination rate and viability. (A) The germination rate of *C*. *spinosa* seeds with different DRTs. (B) The viability of *C*. *spinosa* seeds that did not germinate with different DRTs. The data were from 3 independent experiments and were analyzed by ANOVA. * *P* < 0.05 compared to the control group.

Compared with the control group, the DRT of 0–70 h remarkably enhanced the *C*. *spinosa* seed germination rate (Fig 7A). As shown in Fig 7B, no significant difference in seed viability was found among the four groups. These results suggested that passage through the guts of lizards may enhance the success of *C*. *spinosa* seed germination and exert no effect on seed viability.

### Fecal deposition and new seeding sites

In our study area, we found that the habitat selection patterns of seedlings and lizard feces were similar. Both seedlings and lizard feces were randomly selected from deadwood with a selection indices of E_*i*_ = 0.09 and E_*i*_ = 0.1, respectively. The habitats of *A*. *sparsifolia* and *Z*. *fabago* had high selection indices, showing that both seedlings and lizard feces were preferred. The habitats of *Calligonum spp*., dead grass, *K*. *caspia* and clearings were presented as negative selection preferences (Table 1). In addition, lizards did not deposit feces in the various microhabitats in proportion to their availabilities at the study site (χ^2^ = 181; d.f. = 12; *P* < 0.001). Approximately 21.52% of all lizard feces collected were deposited near *A*. *sparsifolia* and *Z*. *fabago*, which can potentially be used as nurse plants. This type of microhabitat represents only 2.95% of the ground cover at the study site.

**Table 1:** Microhabitat selection of feces and seedlings.

Research object	*i*	*p*_*i*_(%)	*r*_*i*_(%)	*W*_*i*_	E_*i*_	Selection
Seedling	*Capparis spinosa*	20.05	50.00	0.14	0.05	RS
	*Alhagi sparsifolia*	1.11	8.33	0.42	0.54	PS
	*Zygophyllum fabago*	1.84	8.33	0.25	0.34	PS
	*Calligonum spp*	2.56	0.00	0.00	-1	NS
	*Karelinia caspia*	1.67	0.00	0.00	-1	NS
	Deadwood	6.25	16.67	0.15	0.09	RS
	Dead grass	14.95	8.33	0.03	-0.60	NS
	Clearings	51.57	8.33	0.01	-0.87	NS
Feces	*Capparis spinosa*	20.05	42.44	0.08	-0.20	NS
	*Alhagi sparsifolia*	1.11	11.05	0.39	0.51	PS
	*Zygophyllum fabago*	1.84	10.47	0.22	0.28	PS
	*Calligonum spp*	2.56	1.74	0.03	-0.65	NS
	*Karelinia caspia*	1.67	5.23	0.12	-0.01	RS
	Deadwood	6.25	24.42	0.15	0.10	RS
	Dead grass	14.95	1.74	0.00	-0.93	NS
	Clearings	51.57	2.91	0.00	-0.97	NS

Note: RS = random selection, NS = negative selection, PS = positive selection.

## Discussion

In this study, we found that longer MRTs of *C*. *spinosa* seeds in the digestive tracts of lizards enhanced the permeability of the seed coats, which promoted fast water uptakes, broke seed dormancy and increased the seed germination rates. Moreover, by depositing intact and viable seeds onto safe potential recruitment sites, *T*. *roborowskii* is considered to be a legitimate seed disperser of *C*. *spinosa*.

### Retention time

DRTs are reported to directly impact food digestibility, which probably depends on the gut volumes and food intakes of animals [31]. A number of studies have shown that DRTs are quite different among diverse animals; the DRT of monkeys was found to be 16–25 h [32], that of tortoises was 6–28 days [13], that of frugivorous birds was 15–45 minutes [33], and those of ruminants including cattle [34], elands [35] and horses [36] were generally 70–90 h, 45–70 h and 25–35 h, respectively. In this study, feeding experiments were conducted to examine the effects of age and food type on food intake rates and DRTs using captive *T*. *roborowskii* specimens. We found that both adult and juvenile lizards had higher mealworm intakes than *C*. *spinosa* intakes.

The addition of nontoxic small glass beads to food has been a successful method for determining the digestion retention times of turtles [11] and mammals [37], even in the field [38]. Because the fresh fruits of *C*. *spinosa* were directly used as plant-based foods (the seeds could not be labeled when fed multiple times), uniform feeding was adopted for comparison with animal-based food. Although there are some shortcomings to this method, we believe that the results still have important reference value. Due to the uncertainty of individual feeding in the wild, there may be long intervals between meals. In addition, the results we obtained in this study can serve the purpose of comparing different DRTs between animal and plant-based foods under the same conditions.

Due to differences in the morphology and physiology of the digestive systems of frugivores, such as mammals, birds and reptiles, the DRTs of seeds in reptiles is longer than those in small mammals and birds [18]. Previous studies have reported that DRTs in lizards usually last 2–4 days [39] or 1–6 days [40]. Here, the transit time of food through the digestive tracts of lizards recorded in our study was nearly 15 days, which is extremely rare in lizards. Animals function as a key biotic vector of seeds, and the scale of the seed dispersal increases with the movement rates of animals and the mean seed retention times. Although lizards that feed on different types of food share very similar TFAs and TLAs, there are considerable differences in the MRTs. As a plant-based food, the TFAs of *C*. *spinosa* seeds were generally 42 h (adult) and 48 h (juvenile) after ingestion, and the TLAs were recorded after 384 h (adult) and 318 h (juvenile), resulting in > 100 h MRTs both in adult and juvenile lizards, which might contribute to the break of seed dormancy and the promotion of seed germination.

### Defecation frequency

As an important part of nutrient cycling and digestive physiology, defecation also plays a vital role in the study of seed dispersal and population size estimations. However, the defecation pattern has not yet been reported in lizards. Our data showed that the excretion of *C*. *spinosa* seeds in lizards occurred approximately 5 days apart, while no obvious defecation temporal regularity existed for animal-based food. According to the excretion rate and cumulative excretion rate of *C*. *spinosa* seeds in lizards, it is most likely that the first and second defecation events play a decisive role in seed dispersal.

### Germination

The capacity of seeds to germinate after ingestion by frugivores is important for understanding the evolution of plant-frugivore interactions [18]. The effect produced on seeds by passage through vertebrate guts varies among plant species. In the case of reptiles, some authors have found that ingestion by lizards either does not influence or negatively affects the seed germination rates of some plant species [41]. It has been reported that crocodile consumption significantly lowers *Leucaena lanceolata* seed germination rates, suggesting that *Crocodylus acutus* is not an effective seed disperser [42]. However, in other plant species, especially those possessing drupe- or berry-type fleshy fruits, higher percentages of germination of lizard-ingested seeds have been reported regularly [43]. Several studies, including ours, have found that the digestive tracts of lizards could enhance seed germination [44]. Our results showed that a DRT of 0–70 h could directly promote the germination rate of *C*. *spinosa* seeds, and, although statistical analysis showed no significant difference between other gut-passed seeds and the control group, the germination rates after DRTs of 72–120 h (9.28%) and 120–360 h (8.84%) were higher than those of the control group (2.92%).

### Water uptake and seed coat

The penetration of seeds can be improved by damage caused by an animal’s digestive system, and these improvements can promote seed germination [45]. The mechanisms by which the digestive system of an animal stimulates germination might be the softening and scarification of seed coats through the actions of acids and enzymes in saliva and the stomach [46]. The digesta retention time of hard seeds has been reported to be more than 65 h [47], which is consistent with our results. This phenomenon may be an adaptive strategy for animal seed dispersal because longer retention times might contribute to the escape of a seed from the maternal plant, which can help avoid intraspecific competition. Moreover, the physical barrier of the seed coat may be weakened by the grinding of the seed disperser, which may promote seed germination. In our study, the *C*. *spinosa* seeds that passed through the digestive tracts of lizards exhibited higher water uptake abilities and germination rates than the control seeds. The possible reason for this result is that the digestive functions of lizards increased the permeability of the *C*. *spinosa* seed coats, resulting in fast water uptake rates within the first 6 h and consequently promoting seed germination. Our results are consistent with the view of Valido that frugivory (in lizards, birds and mammals) may not only lead to the dispersal of seeds but may also lead to the dispersal of viable seeds, which may germinate and increase plant fitness [10].

In addition, the fecal material surrounding seeds after passage through a digestive tracts has been shown to enhance nutrient availability, germination rates and even seedling establishment. However, the responses may differ depending on the species, and other studies have demonstrated that the acidification of soil has negative effects on seed germination [48]. In the present study, the fecal material surrounding the seeds was removed to avoid the influence of nutrients on seed germination. Therefore, further studies are needed to clarify this issue.

### Fecal deposition and new seeding sites

The directed-dispersal hypothesis asserts the nonrandom arrival of seeds at specific sites where establishment conditions are independently favorable. A previous study showed that directed seed dispersal by mutualistic birds can increase the probabilities of germination and survival by the nonrandom deposition of seeds in suitable microhabitats [49]. The location of seed deposition and especially the distance from the source are two key factors determining what happens to a seed after defecation [50]. Usually, lizards deposit their feces into microhabitats, which contributes to seed germination and seedling establishment [51]. In the present study, both seedlings and lizard feces displayed preferential selection of *A*. *sparsifolia* and *Z*. *fabago*, which indicated that the two habitats were conducive to *C*. *spinosa* seedling establishment. Additionally, 27% of lizard feces were deposited on bare soil near deadwood that is commonly inhabited by lizards. However, this microhabitat represents only 3% of the available ground in the study area. The possible reasons for this result are that the soil below deadwood maintains a higher moisture content than that of the surrounding bare ground, and dew accumulation is observed near deadwood, which further improves the moisture conditions in the absence of precipitation. By enhancing seed germination and depositing intact and viable seeds onto safe potential recruitment sites, lizards act, at least qualitatively, as effective dispersers of *C*. *spinosa*. However, there are still some problems worth exploring in the future. For example, the quantity of seed dispersal needs to be further investigated, and lizards should be tracked and marked individually to clarify the scope and pattern of their activities.

In conclusion, our study is the first to report the digesta retention times of *T*. *roborowskii*. The *C*. *spinosa* seeds that passed through the digestive tracts of lizards with higher germination rates were carried to microhabitats that favor their germination and recruitment. These results suggested that *T*. *roborowskii* is a potential disperser of *C*. *spinosa* seeds.

## Supporting information

S1 File (MP4)

S2 File (MP4)

S3 File (XLS)
